# Supplementary material for: Comparison between Colistin and Polymyxin B in the Treatment of Bloodstream Infections Caused by Carbapenem-Resistant Pseudomonas aeruginosa and Acinetobacter baumannii-calcoaceticus Complex
Source: Antibiotics (Basel). 2023 Aug 15;12(8):1317. doi: 10.3390/antibiotics12081317 (PMC10451820; doi:10.3390/antibiotics12081317)
Supplement: Supplementary file 1 [file antibiotics-12-01317-s001.zip › antibiotics-2527252-supplementary.pdf]

Supplementary Table S1. Combination antimicrobial therapy in patients with *Pseudomonas aeruginosa* or *Acinetobacter baumannii-calcoaceticus Complex* bloodstream infections

Combination therapy	Total	Antimicrobial Therapy	
	N= 259	Polymyxin B, N=212	Colistin, N=47
Ampicillin/sulbactam	11 (4.2)	9 (4.2)	2 (4.3)
Ceftazidime	4 (1.5)	3 (1.4)	1 (2.1)
Cefepime	2 (0.8)	1 (0.5)	1 (2.1)
Piperacillin/tazobactam	7 (2.7)	5 (2.3)	2 (4.3)
Ciprofloxacin	2 (0.8)	1 (0.5)	1 (2.1)
Amikacin	55 (21.2)	37 (17.4)	18 (38.3)
Gentamicin	3 (1.2)	3 (1.4)	0
Ertapenem	11 (4.2)	11 (5.2)	0
Meropenem	182 (70.0)	148 (69.5)	34 (72.3)
Tigecycline	21 (8.1)	16 (7.5)	5 (10.6)
Ceftazidime/avibactam	8 (3.1)	4 (1.9)	4 (8.5)

Results are presented as n (%).

Supplementary Table S2- 30-day mortality according to subgroup analysis.

	Polymyxin B (N=212)	Colistin (N=47)	Hazard Ratio* (95%CI)	P value
Bacteria				0.11
Acinetobacter baumannii	80/165 (48.2%)	22/39 (56.4%)	0.92(0.6 – 1.5)	0.74
Pseudomonas aeruginosa	22 /47 (46.8%)	5 /8 (62.5%)	0.47 (0.2 – 1.3)	0.15
Baseline ICU Admission				0.40
Yes	83/146 (56.8%)	25/38 (65.8%)	1.30 (0.8-1.2)	0.29
No	19/66 (28.8%)	2/9 (22.2%)	0.72 (0.2-3.2)	0.66

ICU, Intensive Care Unit.

*Adjusted for COVID-19 infection, Pitt bacteremia score, Charlson Comorbidity Index and time from bacteremia to active antimicrobial treatment.
